# Case Report: Diagnostic imaging for evaluation of five goats suspected to have congenital macroscopic portosystemic shunts

**DOI:** 10.3389/fvets.2026.1971772

**Published:** 2026-10-27

**Authors:** Dane W. Schwartz, Miguel Saucedo-Gonzalez, Robert Cole, Clifford Berry, Oscar Huertas, Jessica G. Prim, Benjamin C. Benson, Katelyn Waters, Jenna W. Stockler

**Affiliations:** 1 Department of Clinical Sciences, College of Veterinary Medicine, Auburn University, Auburn, AL, United States; 2 Department of Veterinary Diagnostic and Production Animal Medicine, College of Veterinary Medicine, Iowa State University, Ames, IA, United States

**Keywords:** computed tomography, goat, nuclear scintigraphy, portosystemic shunt, trans-splenic

## Abstract

The objective of this case series is to report diagnostic imaging (computed tomography, nuclear scintigraphy and transabdominal ultrasound) for the identification of portosystemic shunts in five Boer (goat) kids. Two kids presented with neurologic abnormalities, while three others were presented for screening for possible macroscopic portosystemic shunts. Trans-splenic nuclear scintigraphy was successfully done in four goat kids. One goat kid was diagnosed with a macroscopic porto-systemic shunt that was further confirmed using computed tomography. Nuclear scintigraphy was used in three goats; these studies were negative for macroscopic portosystemic shunts. Another goat was only diagnosed with a macroscopic porto-systemic shunt using computed tomography alone. In these five kids, computed tomographic angiography (*n* = 2), nuclear scintigraphy (*n* = 4) and ultrasound (*n* = 2) were all valuable for the evaluation of macroscopic portosystemic shunts. With the increasing popularity of pet livestock, owners are seeking an advanced level of veterinary care and diagnostic imaging, that was once considered impractical.

## Introduction

Portosystemic shunts are often based on a diagnosis based on signalment, history, and clinical signs in livestock species. Supporting clinciopathologic abnormalities include hyperammonemia with normal or mildly elevated hepatic enzymes (1). Portosystemic shunts are anomalous vessels that transport portal blood to the systemic circulation, bypassing the liver, resulting in unfiltered blood returning to systemic circulation (2). Both congenital or acquired porto-systemic shunts have been reported in many species including feline, canine, porcine, camelid, equine, bovine, and caprine (3–9). Clinical signs associated with these aberrant vessels include diarrhea, vomiting, neurologic signs, failure to thrive, and hindered growth of young animals (2). Animals may have a chronic history of poor-doing, but overt clinical signs are often identified at or associated with weaning. In ruminants, the transition from a milk-based diet pre-rumination to a high protein diet after weaning increases the metabolic production and need for liver metabolism and detoxification (10). Antemortem diagnosis of a portosystemic shunt is often presumptive, based on history, clinical signs and clinicopathologic abnormalities (10). Definitive antemortem diagnosis can be confirmed with diagnostic imaging.

Transabdominal ultrasonography is an inexpensive, though insensitive diagnostic technique used to visualize the anomalous vessels (11). Experienced veterinary radiologists may have better success with ultrasonography in identifying abnormal vessels than others (12). In dogs and cats, advanced imaging, like computed tomography angiography (CTA) and nuclear scintigraphy are used to characterize the presence or absence of a shunt as well as the anatomy related to an abnormal shunting vessel. CTA has greater diagnostic sensitivity and accuracy when compared to ultrasonography (9, 11, 13). The use of CTA in food animal species is often limited due to financial constraints.

Nuclear scintigraphy is a non-invasive diagnostic technique. This technique uses the injection of a radionuclide into the spleen or rectally (sodium Tc-99 m pertechnetate). The transit of the radionuclide is then captured via the emission of gamma rays which are subsequently captured by a gamma camera and generated into a series of images (13). The impact of portosystemic shunts on animal welfare and productivity supports the need for investigation into affordable diagnostics (10).

Nuclear scintigraphy does require specialized equipment and is potentially more financially accessible when compared to CTA and may provide an affordable diagnostic alternative for livestock species where available. With the increasing popularity of pet livestock, owners are seeking an advanced level of veterinary care, that was once considered impractical. While the use of trans-splenic portal scintigraphy for the evaluation of portosystemic shunts is well documented in different veterinary species, its use in food and fiber producing animals has not been well documented. The objective of this case series is to report on the diagnostic imaging used in five Boer kids that were referred for possible macroscopic portosystemic shunts.

## Case series description

### Animals

This case series describes five Boer kids that originated from one herd located in east-central Alabama. Full signalment information is provided in Table 1. Throughout the manuscript, kids are identified by case number (1, 2, 3, 4, 5). Reported cases include goat kids from 7–12 weeks of age, that were presented to the Auburn University Large Animal Teaching Hospital (AULATH) from March 2023 to April 2025. Two cases presented to AULATH on an emergent basis for an acute history of neurologic dysfunction characterized by ataxia and blindness. The remaining three cases were evaluated for portosystemic shunt screening.

**Table 1:** Signalment information including designated name, age, sex, and weight. Age is provided in weeks and weight is provided in kilograms.

Name	Age (weeks)	Sex	Weight (kg)
Kid 1	12	Female	17.3
Kid 2	7	Male	17.2
Kid 3	7	Female	12.3
Kid 4	7	Male	10.9
Kid 5	7	Female	17.5

In March 2023, a 12-week-old doeling (Kid 1) was presented to AULATH for abnormal behavior, staggering, and development of blindness. There was no familial relation to the other animals presented in this case series.

In April 2025, a 7-week-old buckling (Kid 2) was presented to AULATH for ataxia and head pressing. Subsequently, three additional cases (Kid 3, Kid 4, Kid 5) were presented for portosystemic shunt screening following euthanasia of Kid 2 due to a diagnosis of portosystemic shunt. All four kids were offspring from the same embryo flush and were therefore from the same sire and dam.

### Ultrasonography

For both kids that were presented with neurologic clinical signs (Kid 1 and Kid 2), initial diagnostics included transabdominal ultrasonography using a macro convex variable frequency (3–5 MHz) transducer on a portable ultrasound machine (Sonoscape X5, SonoScape Medical Corp, Centennial, CO). The thorax and lateral abdomen were shaved (#10 blade) to facilitate transducer contact during examination. Both kids were examined standing with manual restraint. No sedation was used. Examinations were performed by a veterinarian that is a diplomate of the American College of Veterinary Radiology (Diagnostic Imaging and Equine Diagnostic Imaging, RCC). A complete abdominal ultrasound was performed in both kids from the right abdomen including transverse and sagittal planes. The liver was evaluated primarily in the 8-11th intercostal spaces. The main portal vein and caudal vena cava were identified and traced within the liver. Color flow doppler was used to evaluate turbulence and blow direction in attempt to identify a portosystemic shunt or signs of a shunt including hepatofugal portal flow or abnormal turbulence in the caudal vena cava.

### Trans-splenic nuclear scintigraphy

Extra label drug use was done with owner consent and complied with provision of AMDUCA and 21 CFR 530. Four of the five kids (Kid 1, Kid 3, Kid 4, Kid 5) underwent trans-splenic nuclear scintigraphy. Each kid was sedated intravenously with 0.05–0.1 mg/kg xylazine (20 mg/mL, Dechra Veterinary Products, Overland Park, KS, USA) and 0.2 mg/kg midazolam (5 mg/mL, Avet Pharmaceuticals, East Brunswick, NJ, USA). The dosage administered was based on patient response. Adequate patient response was determined by lack of patient movement when placed in lateral recumbency, normal heart rate, regular breathing pattern, constriction of the pupil with central position, and no response when initial ultrasonography was performed. Each kid was placed in right lateral recumbency on a table located above the detector head of the gamma camera (Digital Omega NuCam 55 gamma camera by Diagnostic Services, Middlesex, NJ, USA) using a parallel, low energy collimator with a photopeak set to 140 keV (±10% energy window). Transcutaneous transabdominal ultrasonography using a 4–10 MHz microconvex transducer (Canon Aplio i700, Tustin, CA, USA) was used to locate the spleen, where 105 MBq (3 millicurie (mCi)) of sodium-Tc-99 m pertechnetate was injected into the splenic parenchyma using a 22 gauge 3.8-cm-in needle. A dynamic frame-mode acquisition was initiated after successful needle placement and approximately 1–2 s prior to injection. The frame rate was set to 4 frames/s for 4 min for a total of 960 frames. The images were obtained and stored using a 128 × 128 × 16 (word mode depth) matrix. The dynamic data set was then reformatted into 1 frame per second and reviewed visually for arrival of the radionuclide bolus first to either within the liver (normal) or the heart (abnormal) (Figure 1). Based on a summed image, regions of interest were hand drawn around the area of the heart and liver. These regions were then applied to the dynamic data set at sign of initial radioactivity within either the heart or the liver. A shunt fraction was calculated based on radioactive counts detected in the liver and the heart based on the following equation:

**Figure 1:**
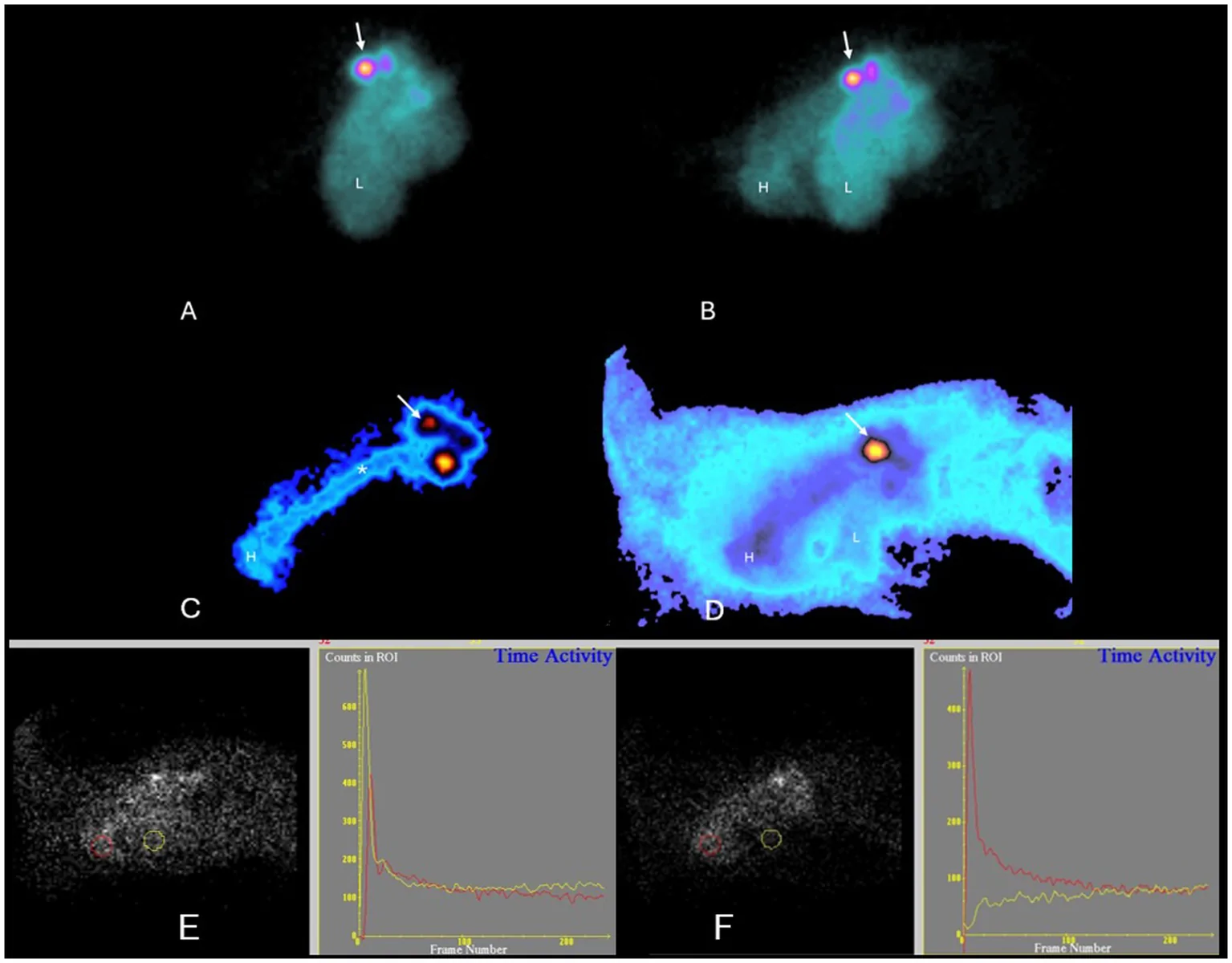
Summed images of dynamic trans-splenic portal scintigraphy using pertechnetate (sodium Tc-99 m pertechnetate) in a normal **(A,B)** and in a young goat with a portosystemic shunt **(C,D)**. In **(A)**, the activity is seen within the liver (L) immediately after injection (white arrow) into the splenic parenchyma. **(B)** is a summed image of the same patient 60 s post A, showing the radioactivity in the liver (L), heart (H), and injection site in the spleen (white arrow). In **(C)**, the activity is seen within the spleen parenchyma (white arrow) as well as the caudal vena cava (white *) and heart (H) bypassing the liver indicating the presence of a portosystemic shunt. In **(D)**, the liver (L), heart (H) and injection site in the spleen parenchyma (white arrow) are labeled for anatomic reference. Dynamic data sets **(E,F)** from a normal goat (Kid 3) **(E)** and from a goat with an intrahepatic portosystemic shunt (Kid 1) **(F)**. In **(E)**, there is a delivery of the radionuclide into the liver prior to the heart whereas in F, there is subsequent delivery of the radionuclide via the systemic circulation into the central circulation and heart prior to the delivery into the liver.

Shunt fraction (%) = [(summed total count of the heart for 7 s)/(summed total count of the heart plus the summed total counts of the liver for 7 s)] x 100 (14)Cole).

At the conclusion of imaging, a reversal agent at 0.1 mg/kg (atipamezole, 5 mg/mL, Zoetis Inc. Kalamazoo, MI, USA) was administered intramuscularly. Each patient recovered without any incident.

### Computed tomography angiography

Kids 1 and 2, were sedated using 0.1 mg/kg xylazine (20 mg/mL Covetrus, Dublin, OH) and either 0.2 mg/kg midazolam (5 mg/mL, 0.2 mg/kg, Hikma) or 2 mg/kg ketamine (100 mg/mL, Zoetis Inc. Germany) to facilitate image acquisition. A two-phase abdominal CTA with contrast medium (Iopamidol 370 Bracco Diagnostics, Princeton, NJ.)was done (Figures 2A–C). The imaging included the use of a helical 128 slice CT scanner (GE medical systems, Milwaukee, WI, USA). The data was stored on a PACS system and reviewed in PACS format. Multiplanar reconstructions (including dorsal and sagittal imaging planes) were produced using routine PACS software (eUnity, Mach 7 enterprises, Burlington, VT, USA) CT images were acquired with 140 kVp and variable mAs in a soft tissue algorithm with 5 mm thick slices reformatted into 0.625 mm slices. Contrast was given via hand injection at 814 mg I/kg of body weight. Post contrast study was obtained immediately following injection of iodinated contrast. The display field of view was just large enough to include the entire circumference of the abdomen.

**Figure 2:**
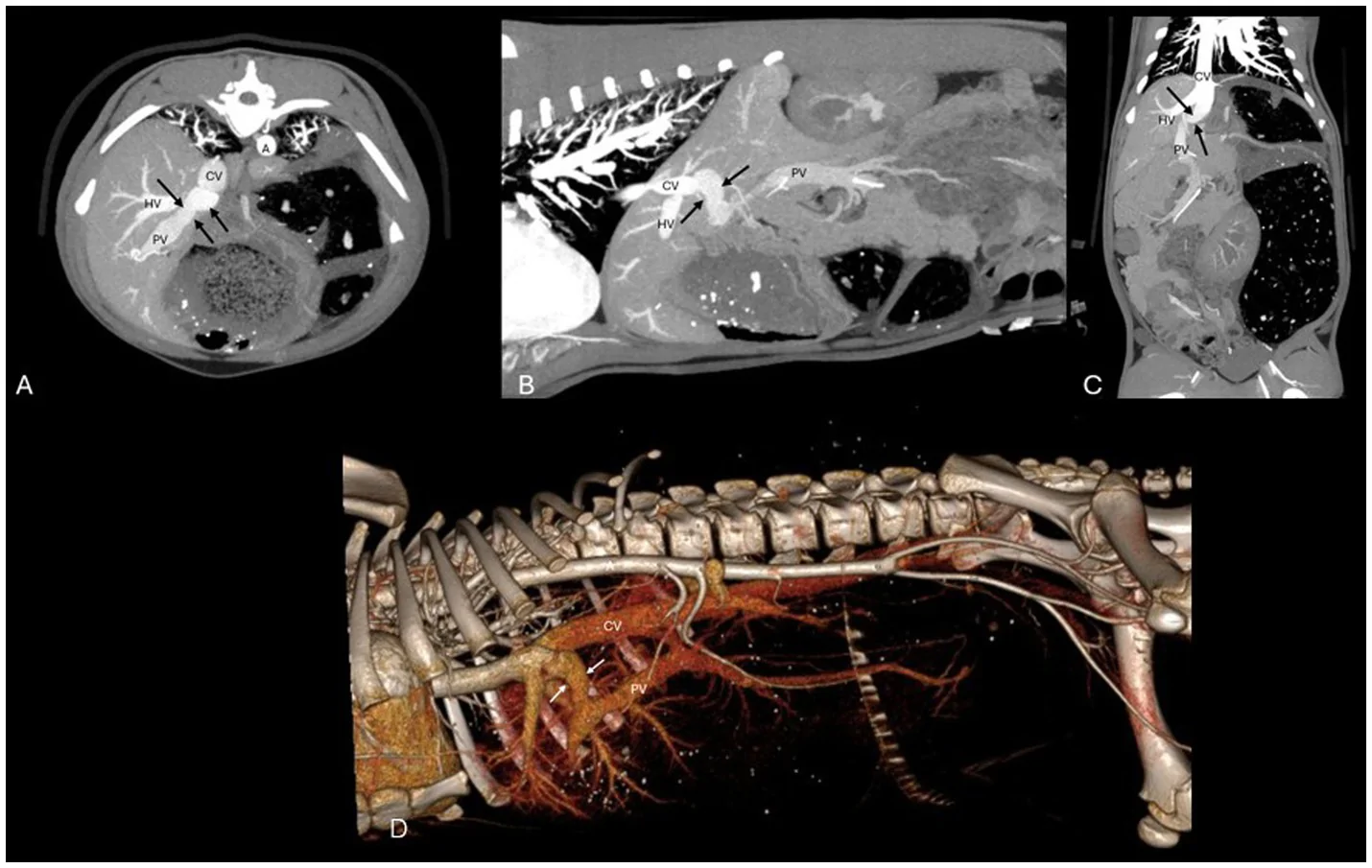
Transverse **(A)**, sagittal **(B)**, and dorsal plane **(C)** post contrast CT images of a young goat (kid 1) with an intrahepatic portosystemic shunt. The shunt is located between the black arrows in all three images **(A–C)**. **(D)** Is a 3D reconstruction of the same patient in **(A–C)**, outlining the anomalous connection between the portal vein and caudal vena cava. CV is the caudal vena cava, PV is the portal vein, HV is a hepatic vein, A is the aorta. The shunt vessel is located between the white arrows.

At the conclusion of imaging, each kid was given a reversal agent at 0.1 mg/kg (atipamezole, 5 mg/mL, Zoetis Inc. Kalamazoo, MI) intramuscular. Each kid recovered without incident.

## Results

### Ultrasonography

No obvious structural abnormalities were observed in Kids 1 or 2. The liver was noted to be normal for size and echogenicity. There were not extra or intra-hepatic shunts identified in either kid. The portal vein and caudal vena cava were normal in appearance on both standard and color doppler evaluation. The imaging of the kids was considered diagnostic by the imager involved in each case (RCC). The cranial and dorsal aspect of the liver was inaccessible due to overlap of the lung.

### Trans-splenic nuclear scintigraphy

For Kid 1, the bolus of activity traveled immediately from the spleen through the splenic portal vein, into the caudal vena cava and then the heart bypassing the liver. This is consistent with a portosystemic shunt. A shunt fraction was calculated and showed to be 91.7%. For the remaining three kids (Kid 3, Kid 4, Kid 5), the bolus of radioactivity traveled from the spleen into the liver circulating within the liver before radioactivity was evident within the heart. There was no evidence of a macroscopic portosystemic shunt (Figure 1). Kid 2 did not have a nuclear scan. The shunt fractions are reported in Table 2.

**Table 2:** Diagnostic imaging (ultrasonography, computed tomography, and nuclear scintigraphy) results for Kids 1–5.

Name	Ultrasonography	Computed tomography	Nuclear scintigraphy
Kid 1	No abnormalities identified	Intrahepatic portosystemic shunt	Portosystemic shunt; Shunt fraction = 91.7%.
Kid 2	No abnormalities identified	Left divisional intrahepatic portosystemic shunt	*
Kid 3	*	*	Portosystemic shunt not detected; Shunt fraction = 2.8%.
Kid 4	*	*	Portosystemic shunt not detected; Shunt fraction = 2.3%.
Kid 5	*	*	Portosystemic shunt not detected; Shunt fraction = 6.6%.

*Indicates diagnostic was not performed.

### Computed tomography angiography

The main finding for Kid 1 was an abnormal branch of the portal vein that traveled dorsally and axially and appeared to join with a branch of the hepatic vein at the level of termination into the caudal vena cava, further confirming and characterizing an intrahepatic portosystemic shunt. Findings for Kid 2 include a large anomalous vessel originating from the left portal branch division which coursed dorsally before entering the left aspect of the caudal vena cava near the level of the left hepatic vein branch. These findings were consistent with a diagnosis of a left divisional intrahepatic portosystemic shunt.

Imaging results for each kid are summarized in Table 2.

### Clinicopathological data

A complete blood count (CBC), blood ammonia, and serum biochemistry analysis were performed on Kids 1 and 2 at hospital admission. Findings are summarized in Table 3. Blood ammonia was not obtained in kids with normal presentation (Kids 3–5) because surveillance imaging was planned, which superseded the need. The most common abnormalities on CBC in Kids 1 and 2 included leukocytosis (24,420 cells/uL; Reference: 4,000–13,000 cells/uL) characterized by neutrophilia (Range: 10,234–18,315 cells/uL; Reference: 1,200–7,200 cells/uL) and anemia (Packed cell volume of 16.7%; Reference range: 22–38%). Abnormal serum biochemical findings included hypoalbuminemia (Range: 2.51–2.54 g/dL; Reference: 2.7–3.9 g/dL), elevated AST (5,070 U/L; Reference: 167–513 U/L), elevated creatinine kinase (64,770 U/L; Reference: 116–464 U/L) and hyperbilirubinemia (total bilirubin) (0.19 mg/dL; Reference: 0–0.1 mg/dL). Consistent hyperammonemia was appreciated in both Kids 1 and 2 (Range: 378–1,284 ug/dL; Reference: 0–60 ug/dL). Cerebrospinal fluid was acquired from the lumbosacral space of Kid 1. Findings consisted of nucleated cells 1/uL (45% macrophages, 45% neutrophils and 10% small lymphocytes) (Reference: <5 cells/uL), red blood cells 1,200/uL (Reference: 0 cells/uL) and protein 16.5 mg/dL (Reference: 0–40 mg/dL). The fluid was clear and transparent.

**Table 3:** Clinicopathologic data (complete blood count, serum blood chemistry, and blood ammonia) results for Kids 1 & 2 on presentation.

Clinicopathologic data	Kid 1	Kid 2
White blood cells (Reference: 4,000–13,000 cells/uL)	24,420 cells/uL	13,830 cells/uL
Neutrophils (Reference: 1,200–7,200 cells/uL)	18,315 cells/uL	10,234 cells/uL
Hematocrit (Reference: 22–38%)	35.7%	16.7%
Albumin (Reference: 2.7–3.9 g/dL)	2.51 g/dL	2.54 g/dL
AST (Reference: 167–513-145 U/L)	5,070 U/L	49 U/L
Creatinine Kinase (Reference: 116–464 U/L)	64,770 U/L	70 U/L
Total Bilirubin (Reference: 0–0.1 mg/dL)	0.19 mg/dL	0.06 mg/dL
Blood Ammonia (Reference: 0–60 ug/dL)	1,284 ug/dL	378 ug/dL

### Necropsy

Necropsy was performed at the Auburn University College of Veterinary Medicine Pathobiology diagnostic services laboratory for Kid 1. Grossly, a portosystemic shunt was not appreciated. Histopathologic abnormalities of the liver included moderate to marked lobular hyperplasia with arteriolar hyperplasia, portal vein hypoplasia, biliary hyperplasia and hepatocyte degeneration. Additionally, histologic abnormalities of the brain included mild multifocal Alzheimer type II astrocytosis. Both findings are consistent with a portosystemic shunt and hepatic encephalopathy.

A standard necropsy was not performed in Kid 2.

## Discussion

This case series describes the use of various diagnostic imaging techniques for the evaluation of kids with the suspicion of macroscopic portosystemic shunts. This report evaluates the use of trans-splenic nuclear scintigraphy as a diagnostic aid and screening tool for the evaluation of portosystemic shunts in the goat. The two animals presented with neurologic disease were diagnosed with an intrahepatic portosystemic shunt using advanced imaging (CTA – both Kids 1 and 2). Trans-splenic nuclear scintigraphy was used as an initial diagnostic aid for the diagnosis of a portosystemic shunt, which was further confirmed and characterized using CTA in Kid 1. The other affected kid (Kid 2) did not have trans-splenic nuclear scintigraphy done, but rather a CTA. Due to confirmation of a portosystemic shunt on the latter kid by CTA, it was elected to perform screening of the clinically normal embryo-transfer siblings (Kids 3–5). Kids 2–5 were from the same embryo flushing but were transferred into different recipients. Nuclear scintigraphy was elected as a screening diagnostic tool in Kids 3–5 due to availability, accuracy and the technique was cheaper at our institution when compared with CTA.

The genetic basis for portosystemic shunts in the dog is not fully understood (14). There is a hereditary association in some purebred breeds, notably the Irish wolfhound (15). A hereditary basis for portosystemic shunts in the goat has been suggested (16). The current cases presented four genetically similar kids, one out of being diagnosed with a portosystemic shunt. There has been no association between goat breed and the incidence of portosystemic shunt (6, 15, 16).

The pathogenesis of hepatic encephalopathy is not fully elucidated. Clinical signs are associated with an increased delivery of neurotoxins in affected animals (1). Neurotoxins, including ammonia and aromatic amino acids, reach the central nervous system due to insufficient liver detoxification or hepatic circulation dysfunction (1). Hepatic encephalopathy is appreciated in cases of hepatotoxins, portosystemic shunting, metabolic diseases affecting liver health (pregnancy toxemia, hypoglycemia), plant toxicosis, salt poisoning, copper deficiency, and trauma (6). In ruminants, microbial conversion of protein to ammonia (NH_3_) is necessary for rumen microbial growth (17). Excess NH_3_ is absorbed into systemic circulation and travels to the liver where it is converted to urea or glutamine and secreted back into the GI tract, kidney, and salivary gland (17). NH_3_ detoxification is essential in the body’s ability to prevent build-up of NH_3_. Excess NH_3_ development is damaging to the brain, leading to increased permeability of the blood–brain barrier and resultant spongy degeneration of the central nervous system (6, 17). Clinical signs associated with hepatic encephalopathy include ataxia, cortical blindness, circling, head pressing, teeth grinding, seizures, anorexia, and generalized weakness (6).

Blood ammonia analysis is a standard test used to assess liver function in large animals. Its use in detecting portosystemic shunts has been described (18). Blood ammonia concentration was not performed on Kids 3, 4, and 5. This is a limitation and would have further enhanced that these animals lacked a portosystemic shunt. This diagnostic was not performed in the normally presenting kids because imaging was utilized (6). Blood ammonia analysis is used at this institution for patients with diarrhea, hepatic dysfunction, portosystemic shunting, and neurological conditions (18).

Cerebrospinal fluid (CSF) analysis is a commonly used diagnostic technique for patients that are presented with neurologic disorders. Acquisition of CSF is routinely performed at the lumbosacral space in the goat (19). Constituents of normal and abnormal CSF analysis have been described (20, 21). In the presented cases, CSF analysis was non-diagnostic due to blood contamination, which is a limitation associated with CSF collection. While blood contamination hindered the true interpretation, the cell count was still within normal limits (20, 21). A normal CSF analysis would be expected in patients with solely portosystemic shunting, although ammonia within the CSF would likely be elevated (10). CSF ammonia testing is not considered part of the routine CSF analysis.

Technologic advancement and operator expertise has made the use of ultrasonography a highly specific and sensitive diagnostic modality for portosystemic shunts in the cat and dog (12, 22). Both extrahepatic and intrahepatic shunts may be identified accurately, though a methodical ultrasonographic examination must be done to reduce false negative shunt evaluations (22). In the goat, the liver is visualized from the 7th – 12th intercostal space (ICS) (23). Imaging of the caudal vena cava and portal vein can be done in the dorsal and ventral 10th and 11th ICS, respectively (23). The entire tracking and visualization of the caudal vena cava and portal vein is difficult, due to super-imposition of the lungs at the 6th and 7^th^ ICS (23). The rumen makes ultrasonographic examination of these structures on the left side of the animal difficult. In the cases presented herein, no abnormalities were appreciated on ultrasonography, despite diagnoses of a portosystemic shunt on advanced imaging. Anatomical differences make the use of ultrasonography challenging for diagnosing portosystemic shunts in goats. In the author’s experience, ultrasound has a high specificity but lacks sensitivity due to the difficulty tracking the vasculature. Alternative cross sectional imaging modalities such as MRI and CTA make visualization and characterization of shunts more achievable.

Nuclear scintigraphy is not solely used for portosystemic shunt diagnosis; other uses include evaluating glomerular filtration rates, liver function, thyroid function, bone metabolism, and mucociliary function (13). Portal scintigraphy is a quick, non-invasive, sensitive and specific imaging technique for the evaluation of portosystemic shunts when compared to other diagnostic modalities such as magnetic resonance imaging. A 140 keV gamma emitting radionuclide (sodium Tc-99 m pertechnetate) is administered to the patient and followed using a gamma camera providing a nuclear portogram. In small animal patients, portal scintigraphy can be done as either per rectal (given as an enema) or trans-splenic (injected into the spleen with ultrasound guidance). In dogs, per-rectal administration of sodium Tc-99 m pertechnetate is diagnostic, though less efficient when compared to trans-splenic portal scintigraphy (12, 26, 27). This results in poorer image quality and necessitates higher radioactive dose administration compared to trans-splenic scintigraphy. When administered into the rectal lumen, sodium Tc-99 m pertechnetate is absorbed from the distal colon into the colonic and caudal mesenteric veins (part of the portal system). When injected into the spleen, it will be absorbed into the splenic portal vein (12). Per-rectal portal scintigraphy is not as effective in the goat due to the presence of the caudal rectal vein (24). The caudal rectal vein drains into the caudal vena cava and can lead to a false positive diagnosis of a portosystemic shunt if the radionuclide is not placed far enough into the colon during per-rectal portal scintigraphy. As a result, trans-splenic portal scintigraphy is the chosen procedure. Limitations of the current study include the small number of cases and not all patients got all three imaging studies, making a direct comparison unavailable. The limitations of each diagnostic imaging test have been reviewed elsewhere (11, 12, 25). In our experience, scintigraphy is an effective test for determining the presence or absence of a shunt. Specialized imaging equipment and some skill level by the experienced user (as is also true for ultrasound and computed tomography) is needed for interpretation of the study.

The authors must emphasize the importance of the discussion between the veterinarian and owner regarding meat withdrawal time and radioactivity prior to performing trans-splenic portal scintigraphy. In food producing animals that are subjected to trans-splenic portal scintigraphy, must, under no circumstances, enter the food chain or be used for consumption. According to the Food Animal Residue Avoidance Databank (FARAD), there is little information regarding pharmacokinetics data in small ruminants and the use of sodium Tc-99 m pertechnetate. Based on other species that have received sodium Tc-99 m pertechnetate, the radioactive half-life is 6 h with a tissue residue up to 17 days. The radioactive half-life refers to the time it takes for half of the administered molecule, in this case sodiumTc-99 m pertechnetate, to decay or no longer be radioactive. While the tissue residue is the time in which radioactive activity is still present after administration of a radioactive substance. Neither of these terms are synonymous with the meat withdrawal time. This is a critical discussion for any show animal as the reproductive impact has not been determined, but the ability to market and sell this animal is limited and/or prohibited. No studies investigating reproductive effects have been published in the ruminant. Additionally, if an animal was administered sodium Tc-99 m pertechnetate, it must be communicated that there is a strict no consumption recommendation. Failure to comply with the recommendations compromises the safety and security of the food supply.

This manuscript is intended to describe the use of trans-splenic nuclear scintigraphy in a subset of goats that presented to the JT Vaughn Large Animal Teaching Hospital at Auburn University. This case series has a small number of animals (*n* = 4) that underwent trans-splenic nuclear scintigraphy, with only one animal being diagnosed via this imaging modality with further characterization using CT scan. Nuclear scintigraphy was successfully used as a screening tool for a portosystemic shunt, however interpretation must be with caution as further confirmatory imaging (CT scan) was not performed in Kids 3–5. Additionally, one animal described in this series did not undergo trans-splenic nuclear scintigraphy but was diagnosed with a proto-systemic shunt on CT scan. This case series is not intended to be used as a validation study of the diagnostic method, but rather a description of how trans-splenic nuclear scintigraphy was used in these cases. The authors recognize that to make claims of the diagnostic utility and accuracy, a larger number of normal and affected (porto-systemic affected) animals is needed. The reader must consider the presented information and lack of a controlled study when interpreting the claims made in this manuscript.

## Conclusion

The presented case series describes diagnostic imaging techniques for the evaluation of portosystemic shunts that are commonly used in small animals (dogs and cats). Increasing popularity of the pet livestock population increases owner curiosity and willingness to perform diagnostics, which would have otherwise been unrealistic in the past. Further studies are needed to understand the meat and milk withdrawal of sodium Tc-99 m pertechnetate and if it can safely be administered to animals intended for market. Additional studies are needed to validate the sensitivity and specificity of nuclear scintigraphy’s ability to identify portosystemic shunts in the goat.

## Data Availability

The raw data supporting the conclusions of this article will be made available by the authors, without undue reservation.

## References

[1] Smith GW Davis JL Malone JB Nagaraja TG Tomlinson JE Dore V. Chapter 33 - diseases of the hepatobiliary system. In: Smith BP Metre DC Van Pusterla N, editors. Large Animal Internal Medicine (6th Edn). St. Louis, MO: Mosby. (2020). p. 921–55.e6.

[2] Berent AC Tobias KM. Portosystemic vascular anomalies. Vet Clin. (2009) 39:513–41. doi: 10.1016/j.cvsm.2009.02.004, 19524792

[3] Tivers M Lipscomb V. Congenital portosystemic shunts in cats: investigation, diagnosis and stabilisation. J Feline Med Surg. (2011) 13:173–84. doi: 10.1016/j.jfms.2011.01.010, 21338942 PMC11148940

[4] Johnson C Armstrong P Hauptman J. Congenital portosystemic shunts in dogs: 46 cases (1979-1986). J Am Vet Med Assoc. (1987) 191:1478–83. doi: 10.2460/javma.1987.191.11.1478, 3693002

[5] van den Ingh TSGAM van der Linde-Sipman JS Berrocal A Vos JH. Congenital portosystemic shunts in three pigs and one calf. Vet Pathol. (1990) 27:56–8. doi: 10.1177/030098589002700108, 2309382

[6] Kinde H Pesavento PA Loretti AP Adaska JM Barr BC Moore JD . Congenital portosystemic shunts and hepatic encephalopathy in goat kids in California: 11 cases (1999–2012). J Vet Diagn Invest. (2014) 26:173–7. doi: 10.1177/1040638713518050 24532697

[7] Ivany JM Anderson DE Birchard SJ Mattoon JR Neubert BG. Portosystemic shunt in an alpaca cria. J Am Vet Med Assoc. (2002) 220:1696–9. doi: 10.2460/javma.2002.220.1696, 12051513

[8] Marçal VC Oevermann A Bley T Pfister P Miclard J. Hepatic encephalomyelopathy in a calf with congenital portosystemic shunt (CPSS). JVS. (2008) 9:113–5. doi: 10.4142/jvs.2008.9.1.113, 18296896 PMC2839106

[9] Iranmanesh M Sato AF Tenney W Brinker EJ Cortez K Mazan M. Computed tomographic angiography of a presumed acquired transhepatic portosystemic shunt in a miniature horse filly. Vet Radiol Ultrasound. (2024) 65:590–5. doi: 10.1111/vru.13401, 38940068

[10] Humann-Ziehank E Bruegmann M Ganter M. Hepatoencephalopathy in a goat: clinical manifestation of an intrahepatic Porto-systemic shunt. Small Rumin Res. (2001) 42:155–60. doi: 10.1016/S0921-4488(01)00238-3

[11] Kim SE Giglio RF Reese DJ Reese SL Bacon NJ Ellison GW. Comparison of computed tomographic angiography and ultrasonography for the detection and characterization of portosystemic shunts in dogs. Vet Radiol Ultrasound. (2013) 54:569–74. doi: 10.1111/vru.12059, 23763372

[12] Haers H Paepe D Vermote K Saunders J Daminet S RIsselada M. Portosystemic shunts in dogs and cats: imaging portosystemic shunts in small animals–ultrasonography, nuclear scintigraphy, computed tomography, magnetic resonance imaging. Vlaams Diergeneeskd Tijdschr. (2007) 76

[13] Huyhn E Huguet EE Berry CR. "Nuclear scintigraphy". In: Atlas of Small Animal Diagnostic Imaging (2023). Berry CR, Nelson NC, Winter MD editor (Hoboken, New Jersey, USA: Wiley-Blackwell), 36–40.

[14] van Steenbeek FG van den Bossche L Leegwater PA Rothuizen J. Inherited liver shunts in dogs elucidate pathways regulating embryonic development and clinical disorders of the portal vein. Mamm Genome. (2012) 23:76–84. doi: 10.1007/s00335-011-9364-0, 22052005 PMC3275728

[15] Meyer HP Rothuizen J Ubbink GJ van den Ingh TS. Increasing incidence of hereditary intrahepatic portosystemic shunts in Irish wolfhounds in the Netherlands (1984 to 1992). Vet Rec. (1995) 136:13–6. 10.1136/vr.136.1.13 7900255

[16] Wilkerson GK Bera MM Holt TN Callan RJ Pandher K. Hepatic encephalopathy in two goat kids with common paternity. J Vet Diagn Invest. (2008) 20:807–11. doi: 10.1177/104063870802000617, 18987235

[17] Tan Z Murphy MR. Ammonia production, ammonia absorption, and urea recycling in ruminants. A review. J Anim Feed Sci. (2004) 13:389–404. doi: 10.22358/jafs/67425/2004

[18] Or M Devriendt N Kitshoff AM Peremans K Vandermeulen E Paepe D . Ammonia concentrations in arterial blood, venous blood, and cerebrospinal fluid of dogs with and without congenital extrahepatic portosystemic shunts. Am J Vet Res. (2017) 78:1313–8. doi: 10.2460/ajvr.78.11.1313, 29076370

[19] Lanier CJ Fish EJ Stockler JW Newcomer BW Koehler JW. What is your diagnosis? Cerebrospinal fluid from a goat. Vet Clin Pathol. (2019) 48:358–60. doi: 10.1111/vcp.12686, 30720882

[20] Schöb LC Gerspach C Stirn M Hofmann-Lehmann R Riond B. Findings related to cerebrospinal fluid and central nervous system disorders in small ruminants—a retrospective study on sheep and goats. Animals. (2023) 14:46. doi: 10.3390/ani14010046, 38200777 PMC10778181

[21] Kaneko JJ Harvey JW Bruss ML. Clinical Biochemistry of Domestic Animals. 6th ed. San Diego, CA: Academic Press (2008).

[22] Santilli RA Gerboni G. Diagnostic imaging of congenital Porto-systemic shunts in dogs and cats: a review. Vet J. (2003) 166:7–18. doi: 10.1016/s1090-0233(02)00304-0, 12788013

[23] Soroori S Raoofi A Vajhi AR Nezami SG. Ultrasonographic examination of the goat liver. Turk J Vet Anim Sci. (2008) 32:385–8.

[24] Olchowy TW Daniel GB Tucker RL Petersen MG. Hepatic vascular anomaly in a calf. Can Vet J. (1992) 33:131–3. PMC1481148 17423949

[25] Zwingenberger A. CT diagnosis of portosystemic shunts. Vet Clin N Am Small Anim Pract. (2009) 39:783–92. doi: 10.1016/j.cvsm.2009.04.008, 19531401

[26] Cole RC Morandi F Avenell J Daniel GB. Trans-splenic portal scintigraphy in normal dogs. Vet Radiol Ultrasound. (2005) 46:146–52. doi: 10.1111/j.1740-8261.2005.00029.x, 15869160

[27] Sura PA Tobias KM Morandi F Daniel GB Echandi RL. Comparison of 99mTcO4− trans-splenic portal scintigraphy with per-rectal portal scintigraphy for diagnosis of portosystemic shunts in dogs. Vet Surg. (2007) 36:654–60. doi: 10.1111/j.1532-950X.2007.00317.x, 17894591

